# Ischemia/Reperfusion-Induced CHOP Expression Promotes Apoptosis and Impairs Renal Function Recovery: The Role of Acidosis and GPR4

**DOI:** 10.1371/journal.pone.0110944

**Published:** 2014-10-24

**Authors:** Biao Dong, Honglan Zhou, Conghui Han, Jufang Yao, Longmei Xu, Ming Zhang, Yaowen Fu, Qiang Xia

**Affiliations:** 1 Department of Transplantation and Hepatic Surgery, Renji Hospital, School of Medicine, Shanghai Jiaotong University, Shanghai, China; 2 Department of Urology, First Hospital of Jilin University, Changchun, China; 3 Department of Urology, The Affiliated School of Clinical Medicine of Xuzhou Medical College, Xuzhou Central Hospital, Xuzhou, China; 4 Animal Facility of Renji Hospital, School of Medicine, Shanghai Jiaotong University, Shanghai, China; 5 The Central Laboratory of Renji Hospital, School of Medicine, Shanghai Jiaotong University, Shanghai, China; University of Pittsburgh School of Medicine, United States of America

## Abstract

Endoplasmic reticulum (ER) stress-induced apoptosis is implicated in a wide range of diseases, including ischemia/reperfusion injury (IRI). As a common feature of ER stress, the role of CCAT/enhancer-binding protein homologous protein (CHOP) in renal IRI has not been thoroughly investigated. We found that IR led to renal CHOP expression, accompanied by apoptosis induction. Renal IRI was markedly alleviated in CHOP^−/−^ mice. Observations from bone marrow chimeras showed that this was based on CHOP inactivation in renal parenchymal cells rather than inflammatory cells. In vivo and in vitro studies demonstrated that IRI induced CHOP expression in both endothelial and epithelial cells, which was responsible for apoptosis induction. These results were reinforced by the observation that CHOP knockout led to improvement of the postischemic microcirculatory recovery. In vitro studies revealed hypoxia-induced acidosis to be a major inducer of CHOP in endothelial cells, and neutralizing acidosis not only diminished CHOP protein, but also reduced apoptosis. Finally, knockdown of a proton-sensing G protein-coupled receptor GPR4 markedly reduced CHOP expression and endothelial cell apoptosis after hypoxia exposure. These results highlight the importance of hypoxia-acidosis in ER stress signaling regulation in ischemic kidneys and suggest that GPR4 inhibitors or agents targeting CHOP expression may be promising in the treatment of renal IRI.

## Introduction

Acute kidney injury (AKI) caused by ischemia/reperfusion (IR) is a common clinical condition in transplantation medicine featuring high morbidity and mortality [1]. In recent years, growing evidence has shown that endoplasmic reticulum (ER) stress is an important participator linking ischemic insult and apoptosis in various cell types [2]. In addition to the mitochondrial and death receptor pathways, ER is an important organelle in perceiving injury or integrating cell apoptotic signals, on which the idea of an ER stress-mediated apoptosis pathway is advanced [3]. In addition to apoptosis, inflammation can also be initiated by ER stress, which is deemed fundamental in the pathogenesis of inflammatory diseases, including IRI [4]. Evidence shows that signaling pathways in the ER stress and inflammation are interconnected through different mechanisms, ultimately leading to tissue damage [5].

CCAAT/enhancer-binding protein homologous protein (CHOP) is a transcriptional regulator induced by ER stress and a key factor in ER stress-mediated apoptosis pathway. CHOP^−/−^ mice have been shown to be resistant to apoptosis in various disease models, including IRI. For example, ischemia-associated apoptotic loss of neurons was decreased in CHOP^−/−^ mice [6]. CHOP-mediated pathway was also demonstrated to exacerbate myocardial IRI by inducing cardiomyocyte apoptosis and myocardial inflammation [7].

As a common feature of ER stress, the role of CHOP expression in renal IRI has not been thoroughly investigated. By using CHOP^−/−^ mice, we found in this study that CHOP protein in renal parenchymal cells played an important role in apoptosis induction and ischemic renal injury.

## Materials and Methods

### Ethic statements

All animal experiments have been conducted according to standard use protocols, animal welfare regulations and the institutional guidelines of Shanghai Jiaotong University School of Medicine and the Regulations for Practice of Experimental Animals (issued by Scientific and Technical Committee, P.R.China, 1988). All the procedures described were approved by the Animal Use and Care Committee of Shanghai Jiaotong University School of Medicine (approval number: SYKX-2008-0050). All surgery was performed under sodium pentobarbital anesthesia. Analgesia used was bupivacaine (0.5%), a long acting local analgesic, immediately after surgery and only once. Several drops of bupivacaine were dripped on the suture line after the muscle layer was closed, and before the closure of skin wound. All these efforts were made to minimize suffering.

### Animals

CHOP^−/−^ mice (C57BL/6 background, stock number: 005530) were purchased from the Jackson Laboratory (Bar Harbor, ME, USA). Male CHOP^−/−^ mice and wild type controls, 8–12 weeks old, about 20–25 g, were used in this study. Mice used in this study were maintained in a specific pathogen free barrier facility at 24°C, 55% humidity and 12 h light/dark rhythm, and had free access to food and water.

### Renal ischemia-reperfusion model (IR)

The surgical procedures were carried out by an experienced investigator with no prior information regarding the previous treatments and genetic background of the animals. Animals were anesthetized with sodium pentobarbital (60 mg/kg body weight i.p.), prepped for aseptic surgery and placed on homeothermic surgical tables (ALC-HTP Homeothermic System, Shanghai Alcott Biotech Co. Ltd, China) to maintain body temperature of 36°C through rectal probe. A warm renal IR model was used as described previously [8], [9], with minor modifications. In brief, following a midline abdominal incision, right nephrectomy was performed. After intraperitoneal injection of heparin (50 U/kg), left renal pedicle was localized and clamped for 25 min using an atraumatic micro-vascular clamp. After inspection for signs of ischemia, animals were covered with surgical dressing to keep stable intraperitoneal temperature. After removal of the clamp, restoration of blood flow was inspected visually. Sham controls underwent same surgical procedures but without vascular occlusion, hereafter were referred to as non-IR controls. Animals were killed 6 h or 24 h after reperfusion by exsanguination, to obtain blood and renal samples for further analyses. Separate groups of mice were observed after IRI and survival was recorded daily. If an animal was considered possibly morbid, the condition of the animal was monitored every two hours. The presence of morbid symptoms was determined by an experienced observer (Jufang Yao) with no prior information regarding the treatments and genetic background of the animals. Animals were considered morbid if they were severely immobile, hunched in posture, experiencing severe hypothermia, and/or unresponsive to noise. After signs of morbidity were detected, death was considered unavoidable and the animal was euthanized via exsanguinations under anesthesia. The animals that survived to 168 hours after reperfusion were euthanized via exsanguinations under anesthesia, and the successful recovery of renal function was confirmed by serum creatinine/blood urea nitrogen analyses.

### Bone marrow transplantation

Bone marrow transplantation (BMT) was performed as described previously [8]. In brief, male recipient mice aged 8–10 wk were lethally irradiated with a total dose of 10.5 Gy (Cobalt-60, 5.5 Gy+5 Gy, with a 4-hour interval). Bone marrow cells were harvested from male donor aged 8–10 wk. Two hours after irradiation, the recipient mice received bone marrow cells (1×10^7^) by tail-vein injection. Renal IR procedures were performed 30 days after BMT.

### Laser doppler flowmetry (LDF) monitoring in kidneys

The procedures of renal tissue blood perfusion monitoring were as described previously [8], [9]. In brief, a pO2/Flow BareFibre sensor, which was connected to an OxyLab LDF instrument (Oxford Optronix, UK), was inserted into renal outer medulla to enable monitoring of continuous microvascular blood flow. The measurement began 30 minutes after the probe was inserted and continued for 10 minutes with the core temperature maintained at 36°C. The values of ten minutes collected by the probe were expressed as a mean value of blood perfusion units (BPU) over the period of observation. Baseline renal blood flow was obtained by monitoring the microvascular blood flow in the right kidney before IR or sham procedures. After the initiation of reperfusion, the blood flow in the left kidney was measured at the indicated time points, and the mean value versus the baseline result was defined as relative renal perfusion.

### Biochemical analyses

Arterial blood was collected by direct puncture of arteriae aorta. Serum creatinine (Cr) and Blood urea nitrogen (BUN) levels were measured with a standard clinical automatic analyzer (Siemens Dade behring dimension xpand).

### Histology and Histomorphological Scoring of Acute Tubular Injury

Kidney tissues were fixed in 10% neutral buffered formalin overnight, dehydrated, embedded in paraffin and sectioned at 3 µm. For histological analysis, sections were stained with Periodic Acid-Schiff (PAS). Samples were analyzed for tubular cell necrosis, tubular dilation, intratubular cell detachment, and cast formation (original magnification ×200) and were all evaluated in a blinded manner by a nephropathologist. Abnormalities were graded by a semiquantitative histomorphological scoring system from 0 to 4, as described previously [8], [9]. At least 3 fields per section were evaluated.

### Polymorphonuclear leukocyte infiltration (MPO activity)

Renal sections were processed for immunohistochemical localization of myeloperoxidase (MPO, polyclonal rabbit antibody; Novus Biologicals, NBP1-42591), and were then visualized with diaminobenzadine (DAB) and counterstained with hematoxylin. Polymorphonuclear leukocyte (PMN) infiltration was scored semiquantitatively on a scale of 1 (none) to 4 (severe), as described previously [8], [9].

### Terminal deoxynucleotidyl transferase-mediated 2′-deoxyuridine 5′-triphosphate nick-end labeling assay (TUNEL)

Apoptotic cells in formalin-fixed, paraffin-embedded kidney tissue sections were identified with ApopTag Fluorescein In Situ Apoptosis Detection Kit S7110 (Chemicon International), according to the manufacturer’s protocol. Cells with nuclear positive staining by fluorescent antibodies for DNA fragmentation were visualized directly by a fluorescence microscopy and counted (original magnification ×400). At least 3 fields per section were examined, as described previously [8], [9].

### Immunofluorescence staining

After incubation with primary antibodies for anti-CHOP (1/100, Novus Biologicals, Littleton, USA) and CD31 (1/50, Wuhan goodbio technology CO., Wuhan, China) overnight at 4°C, the paraffin-embedded renal sections were washed with PBS for three times and incubated with fluorescein isothiocyanate (FITC) conjugated secondary donkey anti-rabbit IgG (1/200, Wuhan goodbio technology CO., Wuhan, China) and cyanin 3 (Cy3) conjugated secondary donkey anti-goat IgG (1/300, Wuhan goodbio technology CO., LTD, Wuhan, China) for 1 h at room temperature in a darkened humidified chamber. Finally, the preparations were washed with PBS and mounted with fluorescent mounting medium containing 4′, 6-diamidino- 2-phenylindole (DAPI) (Beyotime Institute of Biotechnology, Shanghai, China). Each section was observed under a confocal laser scanning microscope at a magnification of ×400.

### Cell culture and treatment

Human umbilical vein endothelial cells (HUVECs), as well as supplier’s recommended medium and supplement were purchased from ALLCELLS (Emeryville, California, USA). Human proximal tubular cell line (HK-2) was acquired from the American Type Culture Collection (ATCC, Manassas, VA, USA). Cells were cultured according to the suppliers’ instructions. For HR treatment, cells were distributed onto 6-well flat-bottomed plates at a concentration of 5×10^5 ^cells/2 ml/well, and incubated overnight to allow cell adherence. Then cells were exposed to hypoxia (1% O_2_) for 4 hours before went back to normoxic condition. In the pH treatment for HUVECs, immediately after hypoxic treatment was over, the media pH was adjusted to be around 7.4 by using NaOH (1N). Cells were collected at 6 h after reoxygenation. In separate studies, HUVECs were subjected to acidosis, where media pH was adjusted by HCl (1N) to be around 6.4. In some experiments, 8-bromoadenosine 3′:5′-cyclic monophosphate (8-bromo-cAMP, Sigma-Aldrich, USA) was added into the culture media of HUVECs at a final concentration of 500 µM. 3 h, 6 h and 12 h later cells were collected and subjected to immunoblotting analysis.

### Measurement of LDH release

To quantify cell death, the supernatant from cultured HUVECs was collected and lactate dehydrogenase (LDH) concentration was measured by colorimetrically using the CytoTox 96 nonradioactive cytotoxicity assay (Promega, Madison, WI, USA). Results were compared with the maximum release from cells treated with lysis solution and the percentage of LDH release was calculated accordingly.

### siRNA interference

The siRNA duplexes targeting CHOP, GPR4, as well as nontarget scramble siRNA duplexes were provided by Invitrogen (Life Technologies Corporation, NY, USA). CHOP-specific siRNA sequences were: GCUAGCUGAAGAGAAUGAATT and UUCAUUCUCUUCAGCUAGCTT. GPR4-specific siRNA sequences were: CCCUCUACAUCUUUGUCAUTT and AUGACAAAGAUGUAGAGGGTT. Detailed transfection procedures were as described in the previous report [8]. In brief, cells were transfected at a final siRNA duplex concentration of 80 nM in Opti-Mem (Life Technologies Corporation, NY, USA) by using Lipofectamin 2000 (Life Technologies Corporation, NY, USA) in 6-well culture plates for 6 hours. 36 hours after transfection, the cells were subjected to HR treatment.

### Western blot analysis

After the cells or the harvested kidneys were homogenized and lysed with cell lysis buffer, which contained 1 protease inhibitor cocktail tablet per 10 mL of Lysis Reagents (Complete; Roche, Indianapolis, IN). Total protein extracts, after centrifugation at 12,000 g at 4°C for 30 min, was mixed with loading buffer and heated at 99°C for 5 min. Protein concentrations were determined with a bicinchoninic acid (BCA) protein assay kit (Beyotime Biotechnology, China). For western blot analysis an equal amount of protein (60 µg) was loaded in each well and subjected to 12% sodium dodecylsulfate-polyacrylamide gel electrophoresis (SDS-PAGE). Separated proteins were then transferred from the gel to nitrocellulose membranes (Whatman) and blocked with LI-COR blocking buffer for 1–2 h. The membranes were incubated with the primary antibodies overnight at 4°C. The primary antibodies were as follows: CHOP/GADD153 (1∶500, #5554, Cell Signaling Technologies, inc.), cleaved caspase-3 (1∶500, #9664, Cell Signaling Technologies, inc.), β-actin (1∶2000, Santa cruz biotechnology, inc.). After washing primary antibodies with TBS/0.05% Tween-20 for 3 times, the membranes were incubated with appropriate secondary antibodies (1∶10000, LI-COR Biosciences) for 1–2 h at room temperature and then washed again in TBS/0.05% Tween-20 for 3 times. The blot was visualized using an Odyssey infrared imaging system (LI-COR Biosciences). Samples were corrected for background and quantified using Odyssey software. All values were normalized to the loading control and expressed as fold increase relative to control.

### Measurement of intracellular cyclic AMP (cAMP) accumulation

HUVECs were treated with 50 µM 3-isobutyl-1-methylxanthine (IBMX, Sigma-Aldrich, USA) for 30 min, followed by HR treatment. After the treatment, the culture medium was removed. Then 0.1 M HCl 200 ul was added to stop endogenous phosphodiesterase activity and achieve adequate cell lysis. The mixture were boiled for 5 minutes and then centrifuged at 10000 rpm for 5 minutes at 4°C. The supernatant was then collected and the cAMP level was determined by using a Monoclonal Anti-cAMP Antibody-Based Direct cAMP ELISA Kit (NewEast Biosciences Inc., Malvern, USA).

### Transcriptional analysis

RNA of HUVECs was extracted and subjected to quantitative realtime RT-PCR using SYBR Premix Ex Taq (Takara) and values were normalized to β-actin expression. Primer sequences were: TGGCTTTCACCAGCCTCAACTG and AGAAAGCGGAGCAGGTTGTGCA (GPR4); CACCATTGGCAATGAGCGGTTC and AGGTCTTTGCGGATGTCCACGT (β-actin).

### Statistics

All values were expressed as the mean ± SD. Comparisons between two parameters were analyzed by the unpaired Student’s t-test. Statistical significance was set at P<0.05.

## Results

### Renal IRI was accompanied by accumulation of renal CHOP protein

**Figure 1:**
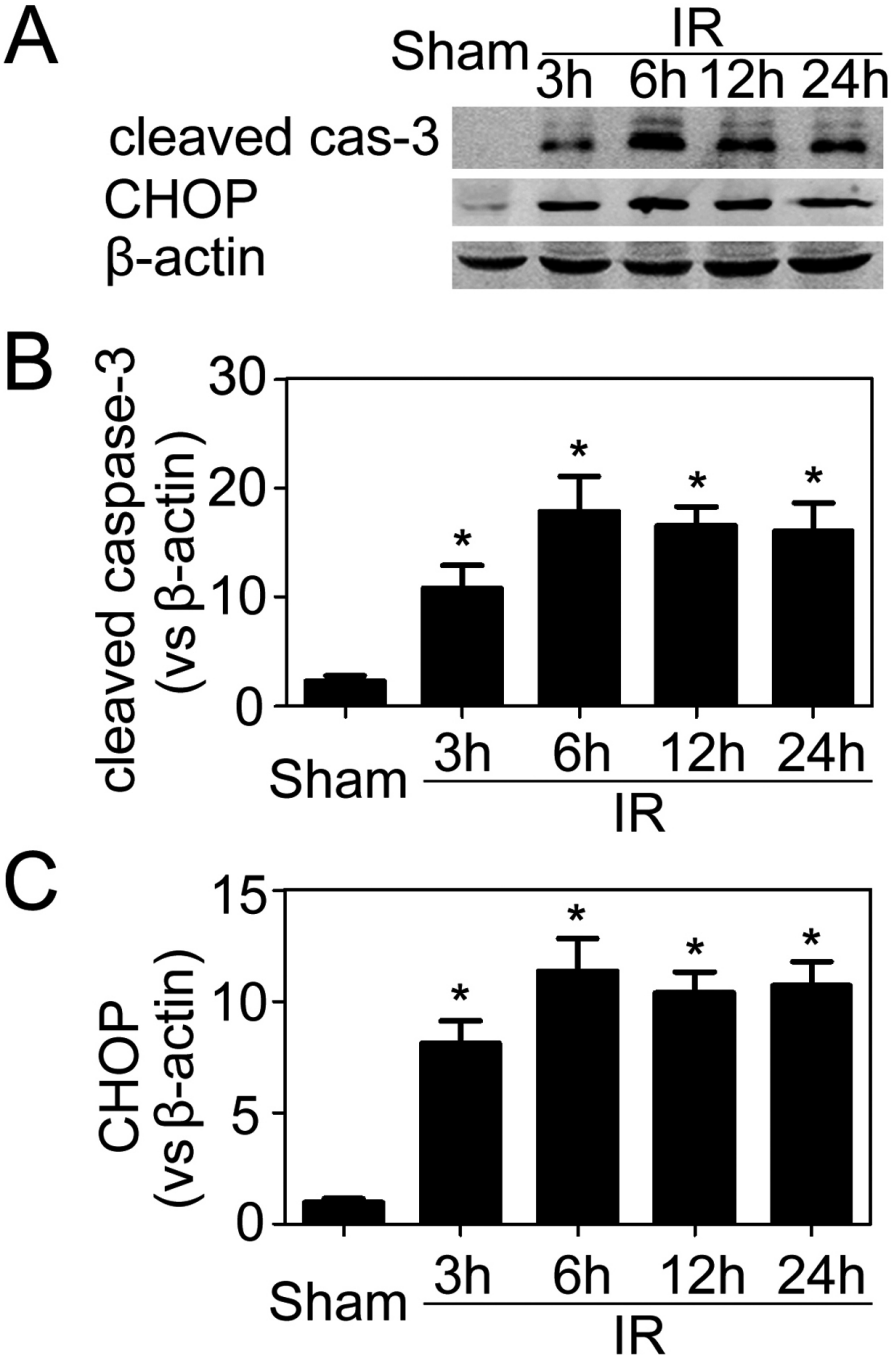
Cleaved caspase-3 and CHOP expression in the post-ischemic kidneys. (A) Representative immunoblotting results from post-ischemic renal samples. (B–C) Quantitative analyses of the relative levels of protein expression. Cleaved caspase-3 and CHOP protein bands were quantified and normalized to β-actin. The mean value obtained from sham-operated mice was arbitrarily defined as 1. There were 6 mice in each group and data were expressed as mean ± SD. *P<0.05 vs. sham-operated controls.

At different time points after the initiation of reperfusion, the IR-challenged kidney was harvested and subjected to immunoblotting analyses for the expression of both CHOP protein and cleaved caspase-3. CHOP was expressed at very low levels in controls but was greatly increased by IR insult. And cleaved caspase-3 expression paralleled CHOP (Figure 1).

### CHOP knockout led to reduced apoptosis and attenuated renal IRI

**Figure 2:**
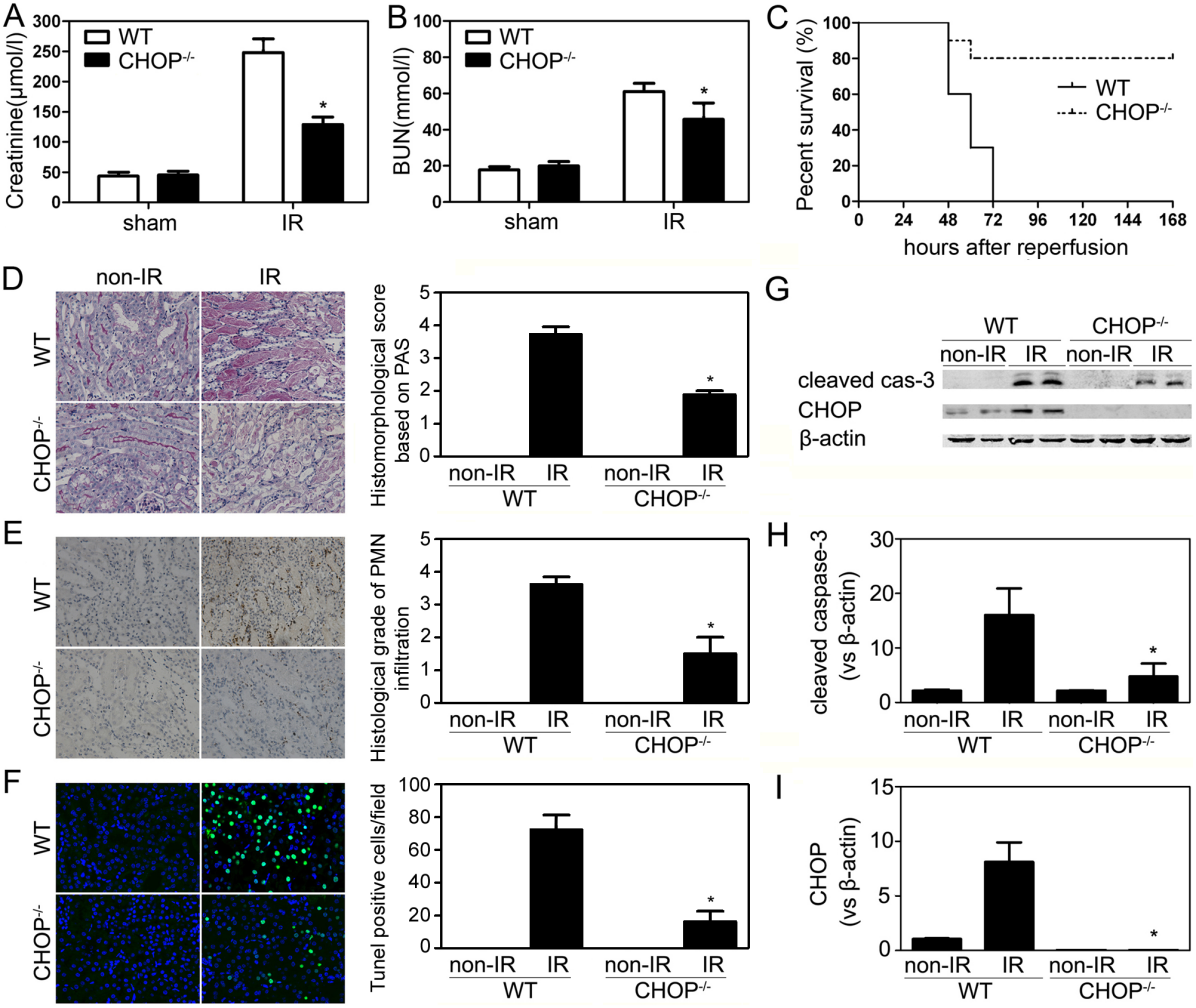
The effect of CHOP inactivation on renal IRI. CHOP^−/−^ and wild-type mice were subjected to right nephrectomy, followed by IR (left kidney) or sham-operation. Serum creatinine (A) and BUN (B) concentrations at 24 h after the initiation of reperfusion were shown (n = 8 per group). (C) Survival of WT and CHOP^−/−^ mice after renal IR operations (n = 20 per group). CHOP knockout led to a significant survival advantage by Kaplan-Meier analysis (log-rank test, P<0.05). (D) Representative PAS-stained sections from post-ischemic kidneys harvested at 24 h (original magnification, ×200). (E) Representative renal MPO staining (original magnification, ×200). (F) TUNEL assay (original magnification, ×400). (G) Representative immunoblotting results of the non-IR right kidneys and the operated left kidneys (6 hours after reperfusion). (H–I) Quantitative analyses of the relative levels of protein expression. Cleaved caspase-3 and CHOP protein bands were quantified and normalized to β-actin. The mean value obtained from non-IR WT kidneys was arbitrarily defined as 1. There were 6 mice in each group and data were expressed as mean ± SD. *P<0.05 vs. WT/IR group.

To evaluate possible differences between CHOP^−/−^ and wild-type (WT) mice in susceptibility to renal ischemic injury, both strains were subjected to renal IR procedures. At 24 h after the initiation of reperfusion, the serum levels of creatinine (Cr) and blood urea nitrogen (BUN) were evaluated and the results were shown in Figure 2. Compared with WT controls, CHOP^−/−^ mice exhibited a significant decrease in both Cr and BUN levels, suggesting attenuated renal dysfunction. Separate groups of mice were subjected to the same IR procedures and survival was observed and recorded for the following 7 days. No deaths occurred in both strains subjected to the sham operation (data not shown). However, no WT mice could survive the IR challenge and all of them died between 48 h and 72 h after IR. By contrast, most CHOP^−/−^ mice survived (80%, P<0.05). The observations were reinforced by histological evidence in PAS staining, MPO activity and TUNEL assay. The evaluation of renal active caspase-3 expression also showed consistent results (Figure 2).

### The alleviated renal IRI in CHOP^−/−^ mice was not a result of CHOP deficiency in inflammatory cells, but in renal parenchymal cells

**Figure 3:**
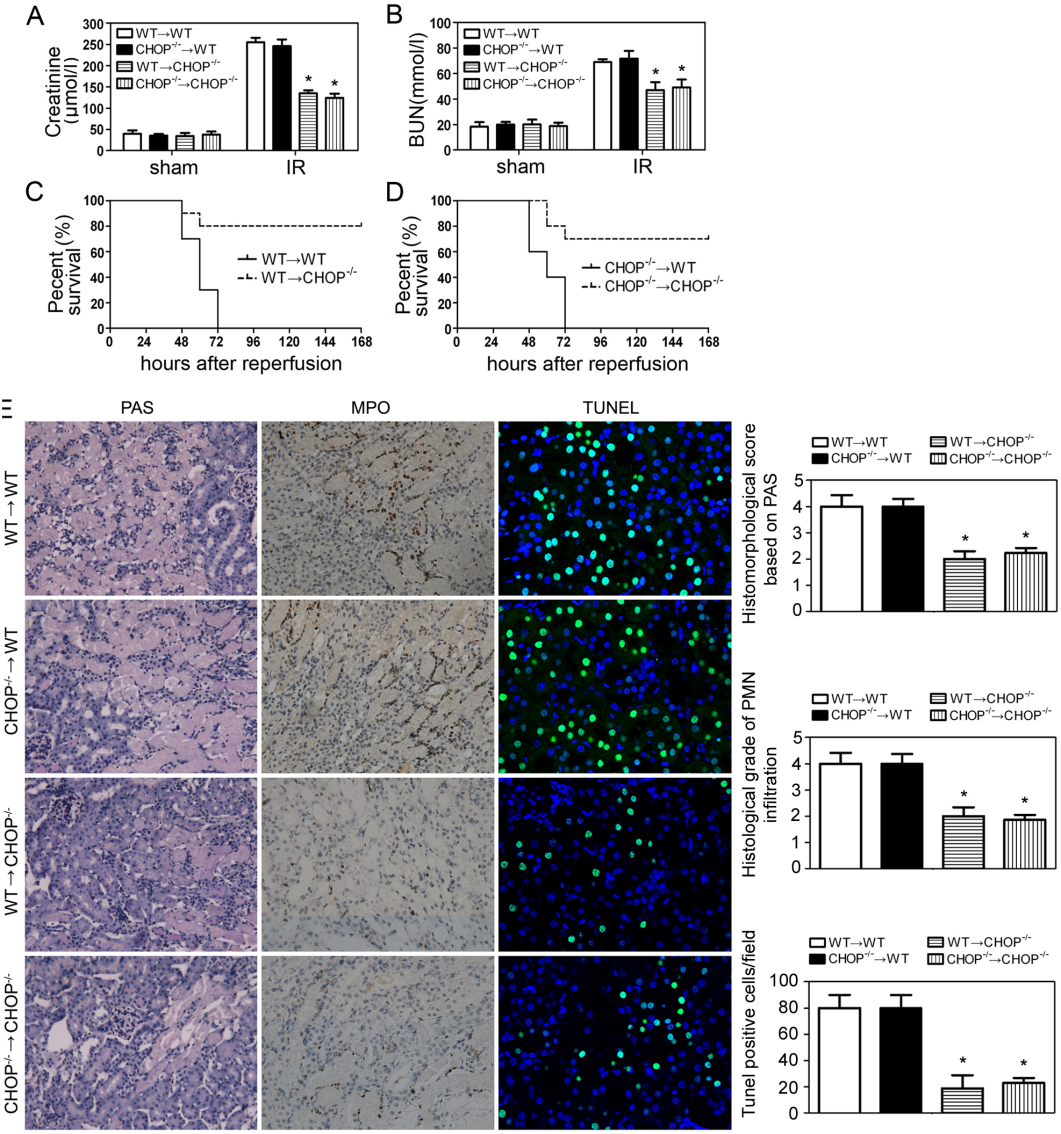
Renal IRI after bone marrow transplantation (BMT). BMT (WT→WT, CHOP^−/−^→WT, WT→CHOP^−/−^ and CHOP^−/−^→CHOP^−/−^) was performed 30 days before renal IR procedures. Blood and kidneys were harvested at 24 h after the initiation of reperfusion. Concentrations of serum creatinine (A) and BUN (B) were measured. Data were expressed as mean ± SD from 6 animals per group. *P<0.05 vs. BMT (WT→WT)/IR group and BMT (CHOP^−/−^→WT)/IR group. (C–D) Survival of BMT mice after renal IR (n = 10 per group). Compared with BMT (WT→WT) group and BMT (CHOP^−/−^→WT) group, BMT (WT→CHOP^−/−^) group and BMT (CHOP^−/−^→CHOP^−/−^) group had a significant survival advantage by Kaplan-Meier analysis (log-rank test, P<0.05). (E) Representative pathological sections and corresponding histological scores of post-ischemic kidneys harvested at 24 h after reperfusion were shown. *P<0.05 vs. BMT (WT→WT)/IR group and BMT (CHOP^−/−^→WT)/IR group.

Although the ultimate result of IRI was the death of renal parenchymal cells, the full development of injury was critically dependent on inflammatory responses. Since CHOP was reported to play a role in inflammatory injury in kidneys [8], we investigated whether CHOP induction in inflammatory cells was involved in IRI, by performing bone marrow transplantation (BMT) 30 days before renal IR procedures. At 24 h after IR serum Cr and BUN levels were evaluated and shown in Figure 3. IRI was significantly attenuated in BMT (WT→CHOP^−/−^), but not BMT (CHOP^−/−^→WT) mice, indicating that CHOP expression in inflammatory cells didn’t play a part in this setting. Survival observations and histological manifestations were consistent with the serum results (Figure 3).

After the studies were finished and the mice were sacrificed, different tissue samples were collected and subjected to PCR analysis, which led to results that could confirm the successful bone marrow engraftment. One of the genotyping figures was shown (Figure S1).

### Renal IR induced CHOP expression in both endothelial and epithelial cells, and endothelial CHOP was induced at a very early stage

**Figure 4:**
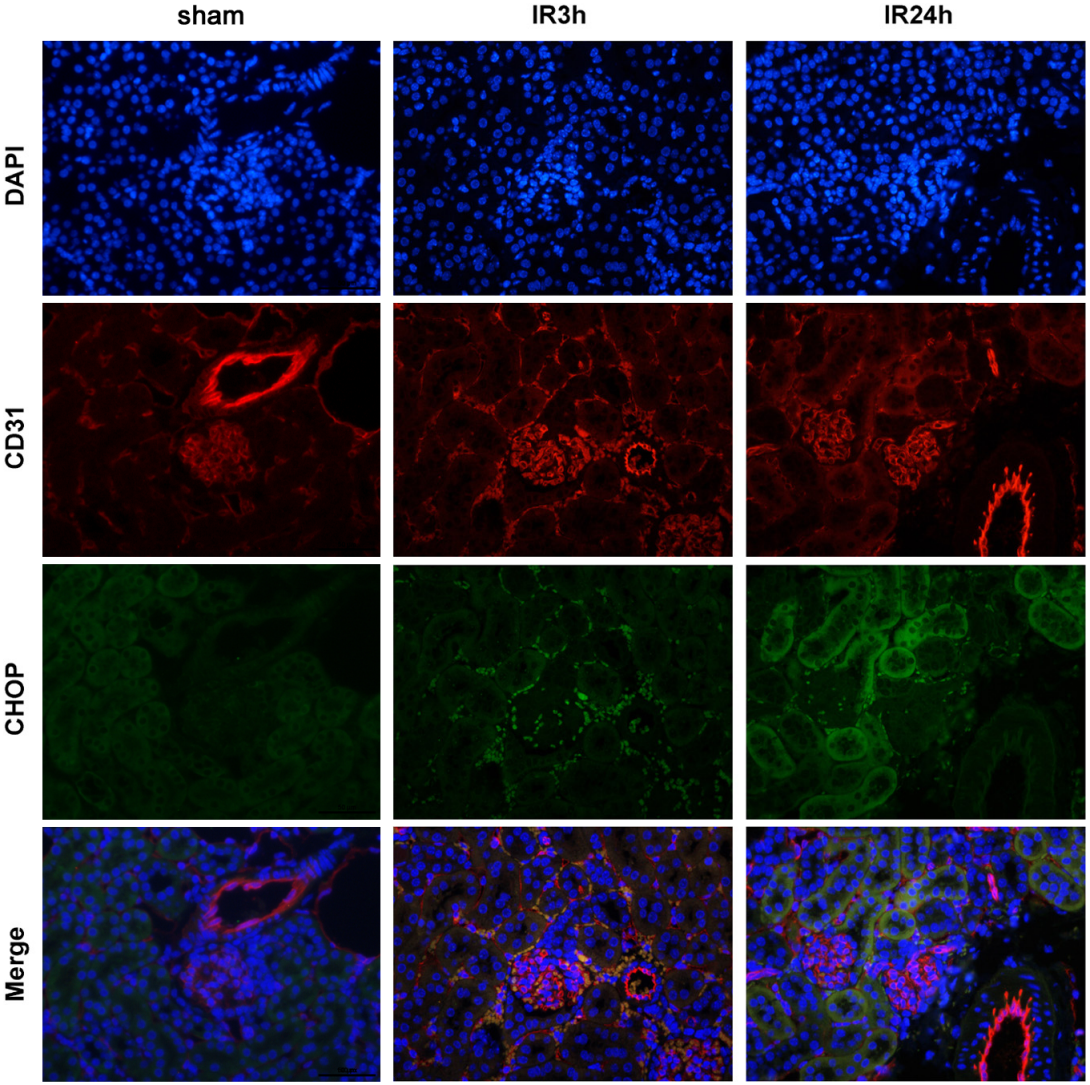
CHOP is prominently present in endothelial cells rather than epithelial cells in the early period after ischemic insult. Representative renal sections from wild-type mice harvested at 3 h and 24 h after ischemic insult (original magnification, ×400). Sections were stained with the indicated antibodies, followed by confocal microscopic analyses.

**Figure 5:**
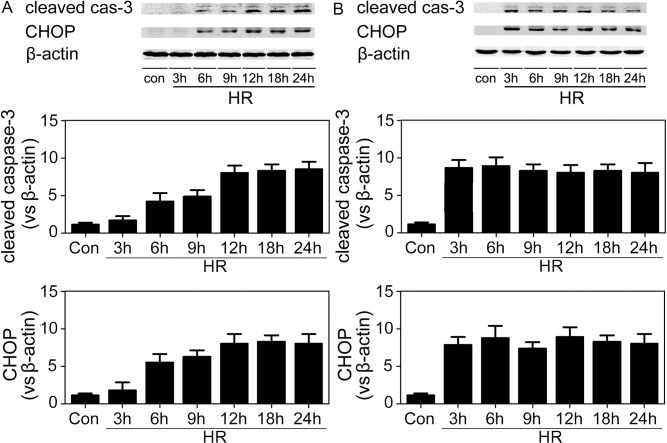
The expressions of cleaved caspase-3 and CHOP in HK-2 cells (A) and HUVECs (B) after HR. The cells were harvested at the indicated time points after exposure to HR and total protein was subjected to immunoblotting analyses. Representative images of 6 separate experiments were shown.

Both renal epithelial cells and endothelial cells are susceptible to ischemic injury and damage to either cell type leads to renal injury. To find out the temporal-spatial pattern of CHOP expression in post-ischemic kidney, we co-stained renal sections with antibodies against CHOP and CD31/PECAM1. Results from these analyses indicated that IR challenge promoted CHOP expression in both endothelial and epithelial cells. Moreover, endothelial CHOP was induced at 3 h after reperfusion (Figure 4). To further dissect the role of CHOP in both cell types, a human renal tubular epithelial cell strain (HK-2) and a human umbilical vein endothelial cell strain (HUVEC) were employed and confronted with HR insult. At different time points after reoxygenation, cells were harvested and total protein was extracted to analyze both CHOP and active caspase-3 expression. As shown in Figure 5, both HK-2 cells and HUVECs accumulated CHOP protein after HR, which was in line with the expression of active caspase-3. The induction of CHOP in HUVECs was very obvious at 3 h after reoxygenation, in keeping with the in vivo results. Thus, these results suggested that CHOP was induced in both renal endothelial and epithelial cells after ischemia/hypoxia challenge and might be involved in apoptosis induction.

### Hypoxia-induced CHOP expression compromised the integrity and function of endothelial cells both *in vitro* and *in vivo*

**Figure 6:**
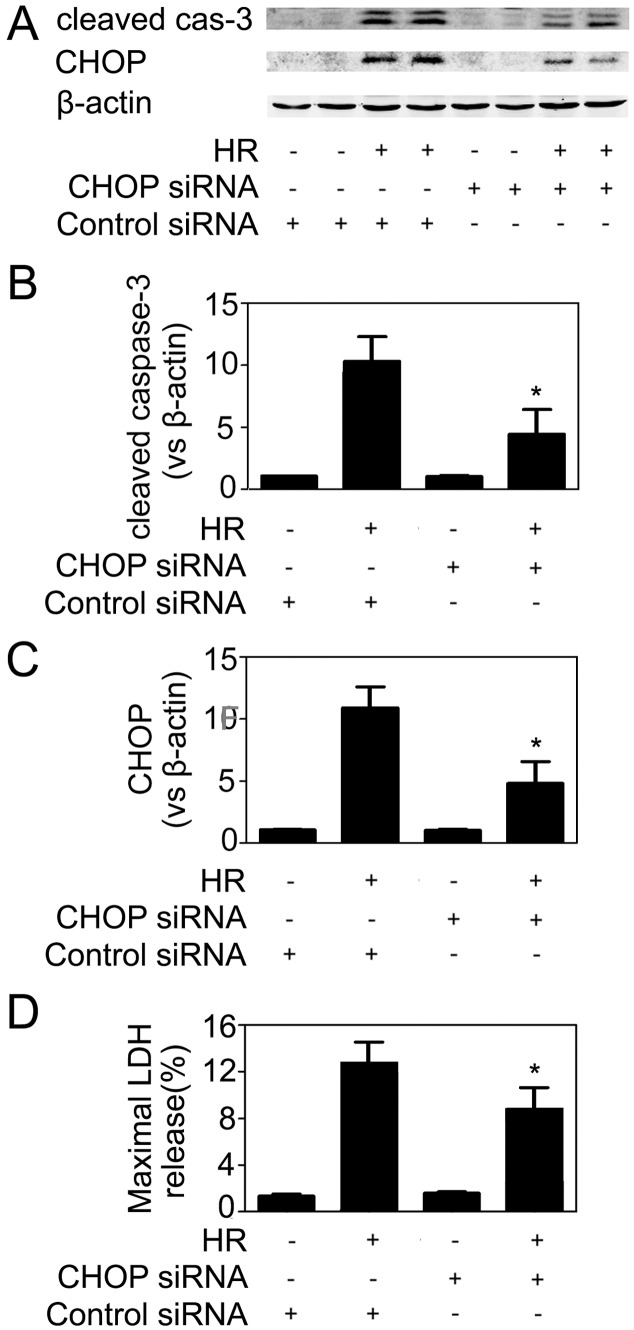
Knockdown of CHOP reduced apoptosis in HR-confronted HUVECs. HUVECs were transfected with a scramble (control) or CHOP-specific siRNA at a final concentration of 80 nmol/l. 36 h later, cells were exposed to 4 h of hypoxia, followed by 6 h of reoxygenation. Then cells were collected and subjected to western blot analysis. Representative images of 6 separate experiments were shown (A). Quantitative analyses of the protein levels. Cleaved caspase-3 (B) and CHOP (C) protein bands were quantified and normalized to β-actin. The mean value obtained from the control was arbitrarily defined as 1. (D) The percentage of LDH release in the supernatant. Data were expressed as mean ± SD from 4–6 separate experiments. *P<0.05 vs. the control siRNA-transfected cells with HR treatment.

A previous study [2] has demonstrated that CHOP played a critical role in HR-induced apoptosis in renal tubular epithelial cells. To investigate the role of CHOP in endothelial cells, we transfected HUVECs with CHOP-specific siRNA before subjected these cells to HR. Knockdown of CHOP expression not only reduced the levels of CHOP protein but also decreased active caspase-3 expression and LDH release (Figure 6), indicating that HR-induced endothelial apoptosis depended on the CHOP pathway, too.

**Figure 7:**
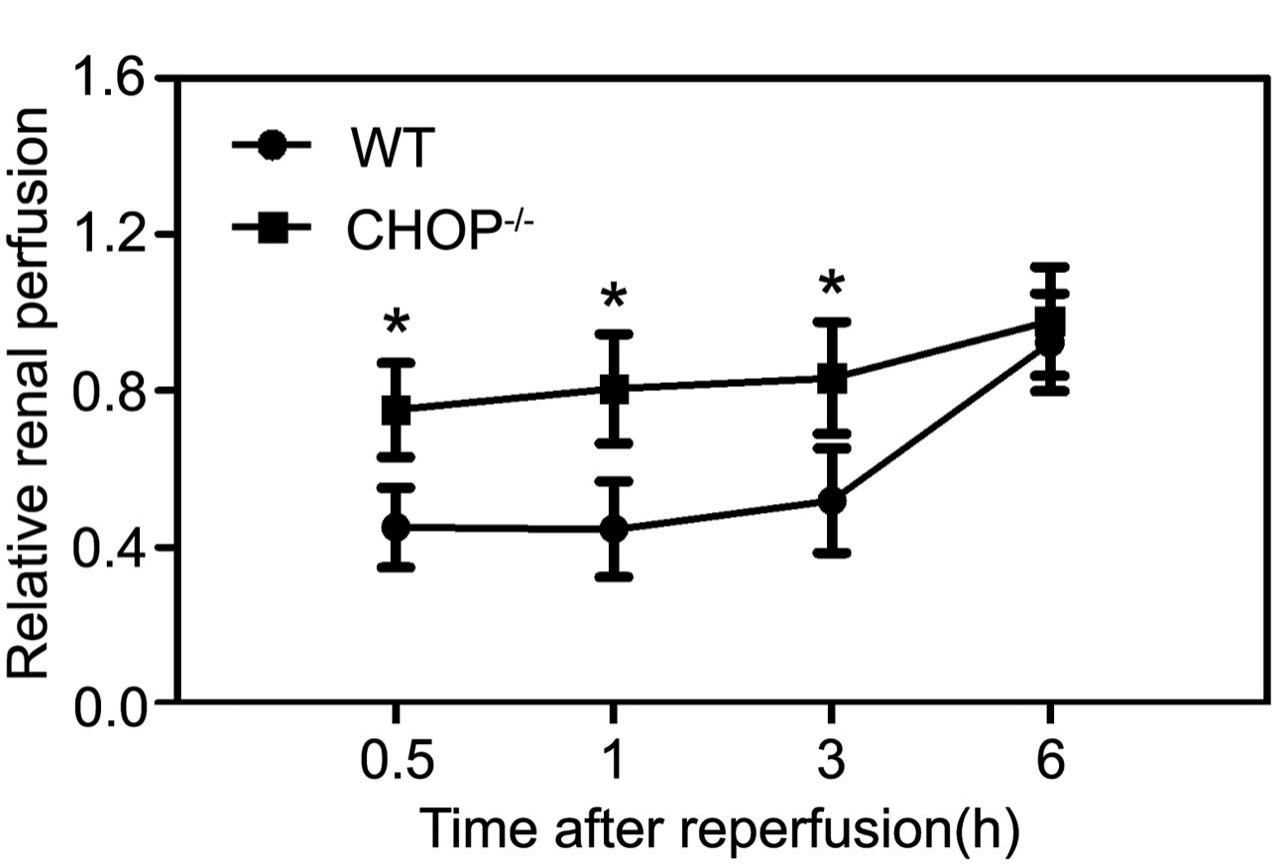
Postischemic renal microcirculatory blood flow recovery in WT and CHOP^−/−^ mice. Baseline renal blood flow was obtained by monitoring the microvascular blood flow in the right kidney. Then the right kidney was removed and the left one was subjected to ischemia. After the initiation of reperfusion, the blood flow in the left kidney was measured at the indicated time points, and the mean value versus the baseline value was defined as relative renal perfusion. Graph showed data acquired from 4–6 independent experiments for each mouse strain at each time point. *P<0.05 between WT and CHOP^−/−^ groups.

To find out whether CHOP inactivation protected endothelial cell function in vivo, we subjected CHOP^−/−^ mice to IR procedures and measured renal blood flow in the outer medulla after the initiation of reperfusion. Baseline microvascular flow, which was obtained from the right kidneys prior to IR operations, was comparable between WT and CHOP^−/−^ mice. As shown in Figure 7, renal blood flow was greatly compromised by the ischemic insult in WT mice. By contrast, CHOP^−/−^ mice displayed much better blood flow recovery.

Taken together, these data suggested that CHOP expression played an important role in hypoxia-induced endothelial cell damage, which led to compromised postischemic renal microvascular perfusion.

### HR-induced CHOP expression in endothelial cells was a result of acidosis

**Figure 8:**
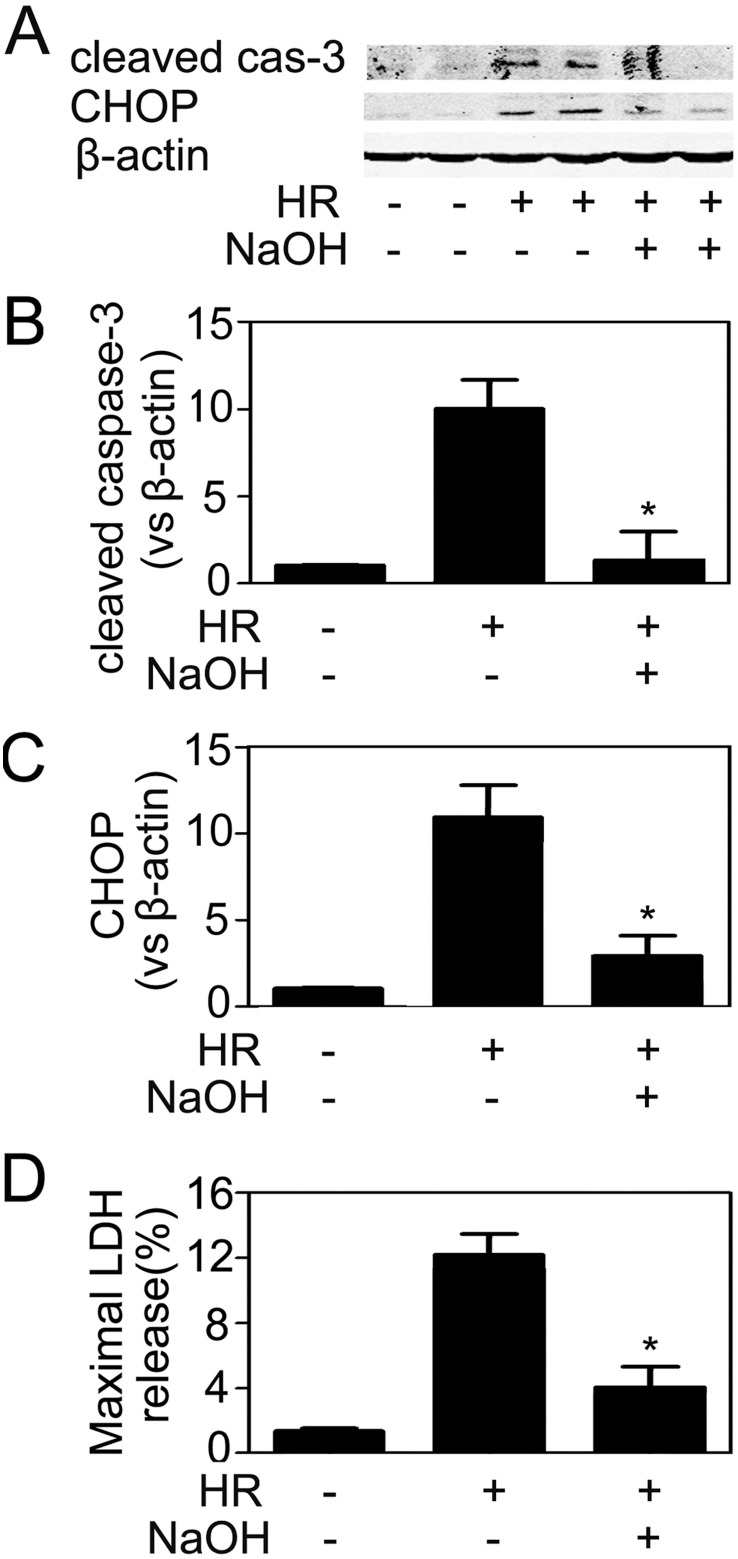
Neutralization of acidosis reduced CHOP expression and apoptosis in HR-treated HUVECs. The pH of culture media was adjusted to 7.4 with NaOH (1N) immediately after hypoxia exposure, followed by 6 h of reoxygenation. Then both cells and supernatants were harvested. (A) The expressions of cleaved caspase-3 and CHOP were determined by immunoblotting assay. (B–C) Quantitative analyses of the relative levels of protein expression. Cleaved caspase-3 and CHOP protein bands were quantified and normalized to β-actin. The mean value obtained from the control was arbitrarily defined as 1. (D) The percentage of LDH release in the supernatant. Data were expressed as mean ± SD from 4–6 separate experiments. *P<0.05 vs. HR-treated group without pH adjustment.

**Figure 9:**
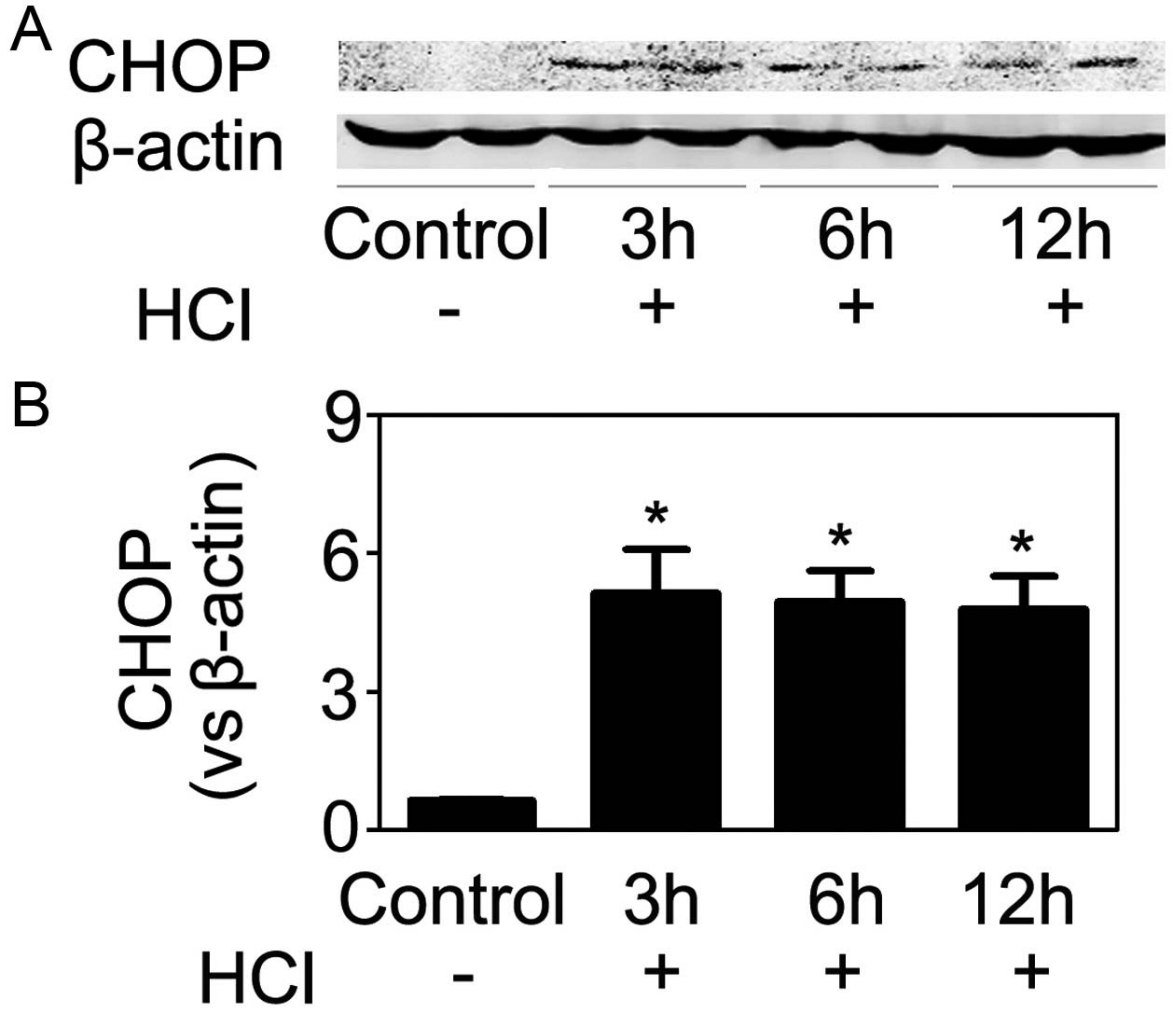
Hydrochloric acid induced CHOP expression in HUVECs. (A) HUVECs were treated with acidic media (pH 6.4), and 3 h, 6 h and 12 h later cells were harvested and CHOP expression was assayed. (B) Quantitative analyses of the relative levels of CHOP expression. The mean value obtained from the control was arbitrarily defined as 1. Data were expressed as mean ± SD from four separate experiments. *P<0.05 vs. the control group.

Although hypoxia is an obligatory consequence of ischemia, it rarely exists alone. Hypoxia in the presence of glucose leads to progressive acidosis of cells in culture. The condition parallels ischemia in vivo, where ischemic tissue becomes rapidly hypoxic and acidotic. In the current study, we found pH value of HUVEC culture medium decreased from the physiological pH (7.4) to the acidic pH (6.4–6.5) after 4 h of hypoxic treatment. To investigate whether acidosis was related to CHOP expression in endothelial cells, we adjusted the pH in culture media with NaOH, immediately after hypoxic treatment to generate a physiological neutral condition (pH = 7.4). 6 hours later, cells were harvested and subjected to immunoblotting analyses for CHOP/caspase-3 expression and supernatant was assessed for LDH release. As shown in Figure 8, the anti-acid additive effectively attenuated CHOP expression. In the meanwhile, apoptosis was reduced when the acidic pH was neutralized. These results indicated that hypoxia caused CHOP expression when there was a simultaneous decrease in extracellular pH. To further validate the conclusion, HUVECs were treated with acidic media (pH = 6.4), which also led to CHOP expression (Figure 9).

Taken together, HR-induced CHOP expression in endothelial cells was a result of hypoxia-induced acidosis.

### HR-induced CHOP expression was based on the proton-sensing GPR4 receptor and its downstream effector cAMP

**Figure 10:**
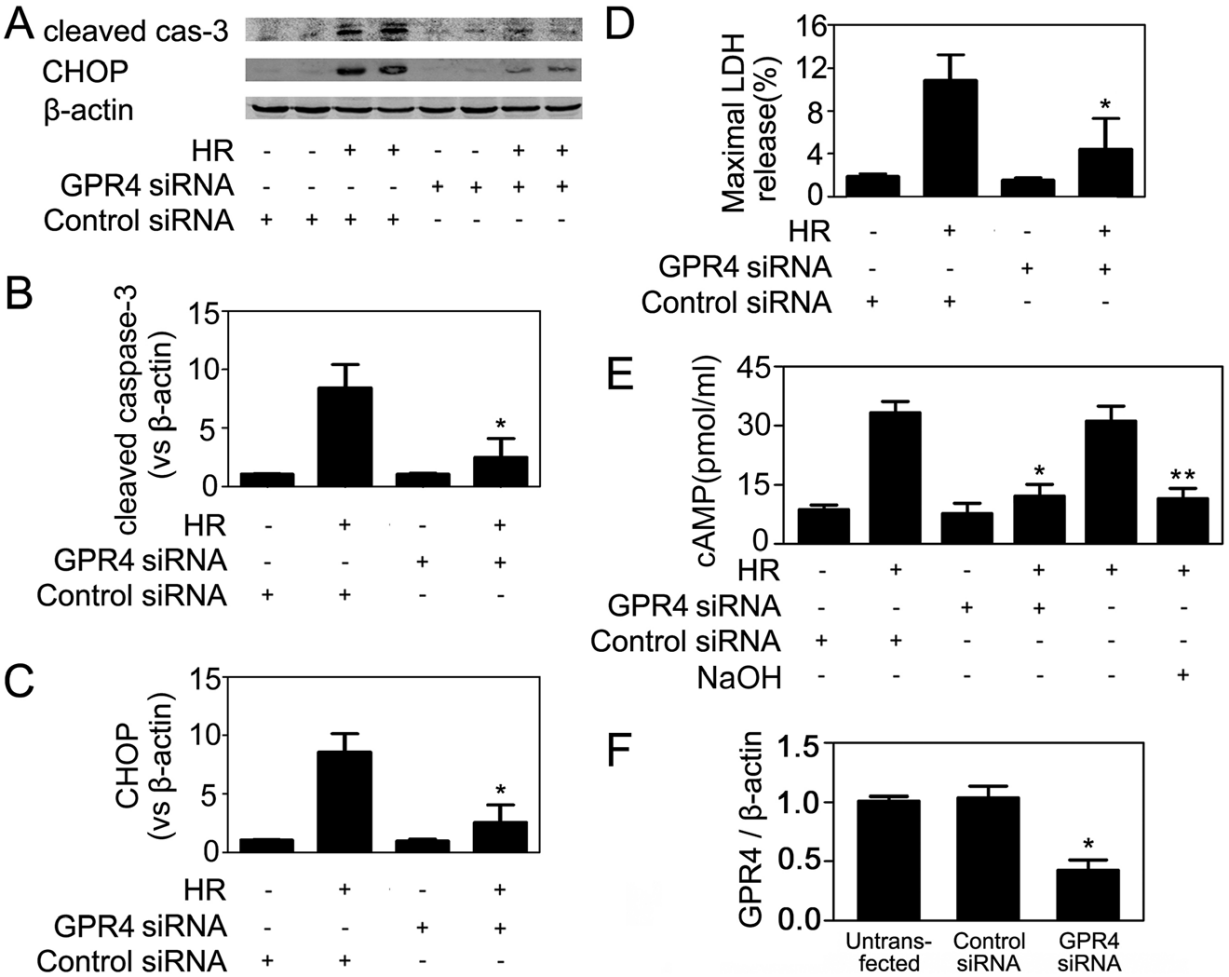
Knockdown of GPR4 reduced cAMP level, CHOP expression and apoptosis in HR-treated HUVECs. (A) HUVECs were transfected with a control or GPR4-specific siRNA. 36 h later, cells were exposed to 4 h of hypoxia, followed by 6 h of reoxygenation. Then cells were collected and subjected to immunoblotting analyses. Representative images of 6 separate experiments were shown. (B, C) Quantitative analyses of the relative levels of cleaved caspase-3 and CHOP expression. (D) The percentage of LDH release in the supernatant. (E) The intracellular cAMP level was determined. (F) Real-time RT-PCR analysis of untransfected HUVECs, scramble siRNA-transfected cells or GPR4 siRNA transfected cells. After transfection and before HR treatment, cells were harvested and mRNA levels of GPR4 were determined. Values were normalized to β-actin. The expression level of GPR4 in untransfected HUVECs was set as 1 and siRNA treatment led to about 60% reduction in target gene expression. Data were expressed as mean ± SD from four separate experiments. *P<0.05 vs. the control siRNA-transfected cells. **P<0.05 vs. the cells subjected to HR, but without NaOH or siRNA treatment.

**Figure 11:**
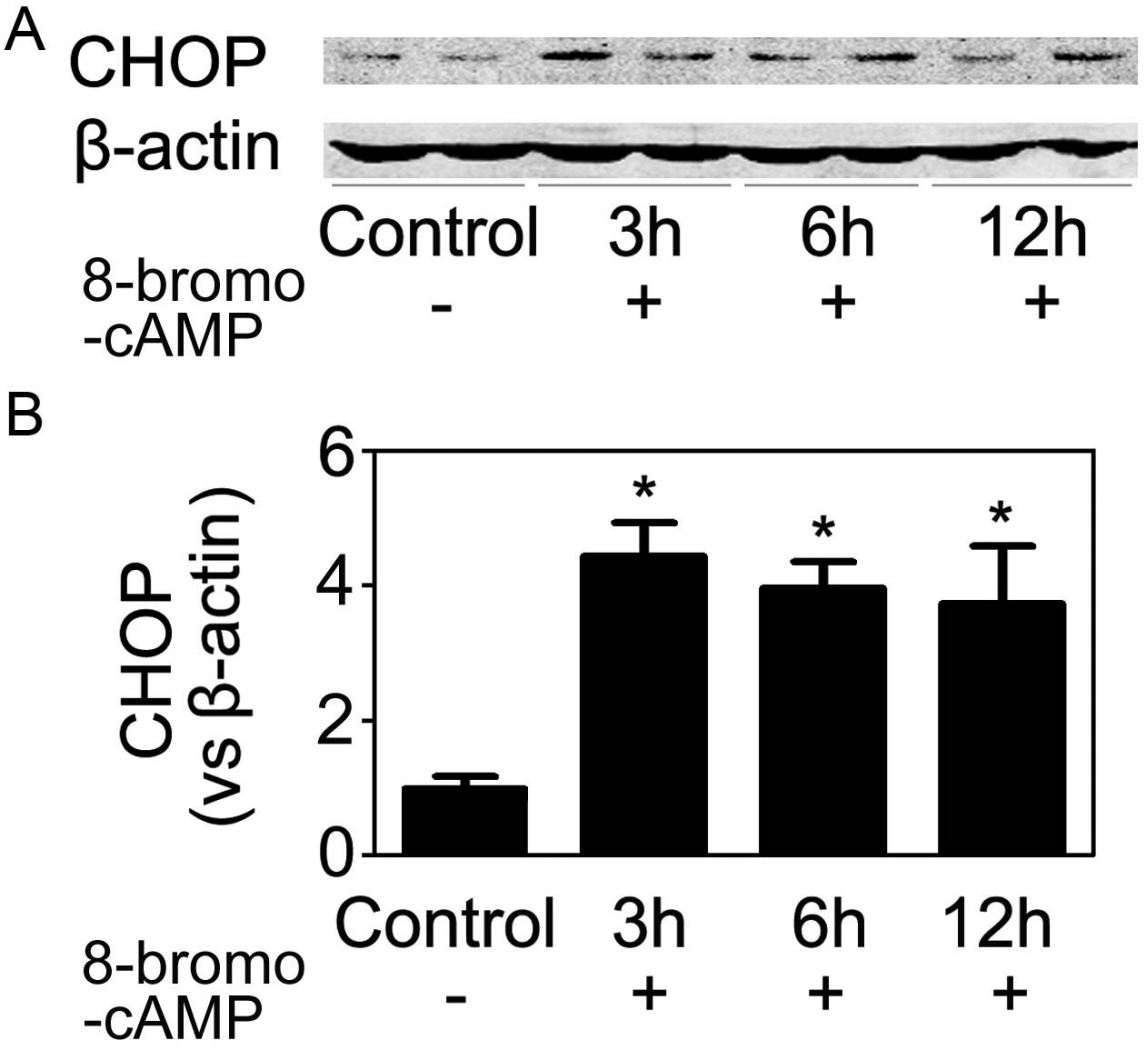
8-bromo-cAMP induced CHOP expression in HUVECs. (A) 8-bromo-cAMP (500 µM) was added into the medium of HUVECs. 3 h, 6 h and 12 h later, cells were harvested and CHOP expression was assayed. (B) Quantitative analyses of the relative levels of CHOP expression. The mean value obtained from the control was arbitrarily defined as 1. Data were expressed as mean ± SD from four separate experiments. *P<0.05 vs. the control group.

The biological effect of hydrogen ion is mediated by a group of “proton-activated” G protein-coupled receptors (GPCRs), among which only GPR4 is relatively abundant in the kidney and acts as a pH sensor in blood vessels. GPR4 activation leads to intracellular production of cAMP, which acts as a downstream effector of GPR4. To dissect the possible molecular mechanism underlying hypoxia/acidosis-induced CHOP expression, we transfected HUVECs with GPR4-specific siRNA, and subjected the cells to HR. Knockdown of GPR4 expression reduced the levels of intracellular cAMP and CHOP protein in HR-challenged HUVECs, and markedly decreased cell apoptosis and death (Figure 10). Finally, 8-bromo-cAMP, a cAMP analog, induced CHOP expression in HUVECs (Figure 11). These results demonstrated that hypoxia/acidosis-induced endothelial CHOP expression was based on GPR4 activation/cAMP accumulation.

## Discussion

Despite the large body of literature linking CHOP protein to many disease conditions, the role of CHOP in cell death and survival is controversial so far. As reported in literature and shown in this report, CHOP is ubiquitously expressed at very low levels but can be greatly increased by stress, which is a hallmark of the unfolded protein response (UPR) and ER stress response. The UPR responds rapidly to ER perturbations under stressful conditions, to restore ER homeostasis and enhance cell survival. However, when UPR is insufficient to handle the prolonged and severe ER stress, the apoptosis program is activated to eliminate the cells that can’t be saved. So UPR and ER stress can go both ways: save it if possible, or kill it if impossible. As a common feature of ER stress, up-regulated CHOP also goes both ways, depending upon context. CHOP is conventionally considered to promote apoptosis. However, more and more evidence shows that CHOP may not be just a killer. Studies employing CHOP^−/−^ mice have demonstrated that CHOP played a protective role in the neurocyte apoptosis and hyperoxia-induced lung injury [10]–[13]. CHOP also protects against LPS-induced inflammation and injury in kidneys [14]. These reports prompted us to seek the role of CHOP in renal IRI, which had not been thoroughly investigated before.

We found that IR quickly induced renal CHOP expression, which lasted at least 24 hours thereafter and correlated well with the expression of cleaved caspase-3. CHOP^−/−^ mice were protected from renal IRI, as evidenced by serum, histological and hemodynamic manifestations, as well as much better survival rate after the ischemic insult. These results highlighted a harmful role of CHOP in the setting of renal IRI.

Acute kidney injury resulted from IR involves multiple mechanisms, among which inflammation plays a key role. So far, the role of CHOP in inflammatory response was controversial [14]–[16]. To find out whether CHOP in inflammatory cells played a role, we conducted bone marrow chimera experiments, which led to results indicating that CHOP inactivation in inflammatory cells didn’t affect the final outcome of renal IR.

IR directly or indirectly attacks tubular epithelial cells and leads to acute tubular necrosis, which accounts for most deleterious effects resulted from IRI. Meanwhile, the integrity and function of endothelial cells are very important to post-ischemic circulatory recovery, which has major impact on epithelial cell function and survival, and thus the final result of renal ischemic injury [8]. It was reported that CHOP was induced in hypoxia-challenged renal tubular epithelial cells and promoted apoptosis [2]. This was in line with our observations. However, the fact that IR also induced CHOP expression in endothelial cells strongly suggested that the vascular system might be another important target of CHOP-mediated damage. As expected, we revealed a causal relationship between CHOP and endothelial cell apoptosis, as well as post-ischemic circulatory dysfunction in vivo. Moreover, the fact that CHOP expression disrupted microvascular homeostasis from the very beginning of reperfusion gave a clue to the important role of CHOP in the pathogenesis of ischemic renal injury, and also, as an ideal molecular candidate for therapeutics of IRI.

Although we inhibited CHOP expression by RNA interference, it was hardly applicable to clinical medicine. Given the fact that CHOP was a common downstream effector of multiple ER stress signaling pathways, it was hard to find effective chemical inhibitors. So it was essential to find out how CHOP was induced in endothelial cells in the setting of HR. Ischemic tissue rapidly accumulated hydrogen ion, which resulted in both extracellular and intracellular acidosis despite humoral and cellular buffers. It was reported that renal tissue pH dropped from 7.2 to 6.5 within five minutes of initiation of ischemia and metabolic acidosis further increased even after the initiation of reperfusion [17]. The molecular mechanisms by which acidosis regulates physiological functions have been documented. The recently discovered family of “proton-activated” G protein-coupled receptors (GPCRs, including GPR4, GPR68, GPR65, etc.) can be stimulated by acidic pH, and functions as pH sensors capable of relaying information about it [18], [19]. Using RNA interference to knockdown the endogenous GPR4 gene expression in HUVECs, we demonstrated that GPR4 played a key role in HR/acidosis-induced CHOP expression and apoptosis induction. A previous study [20] demonstrated that acidosis/GPR4 regulated endothelial cell adhesion through cAMP production and played a role in the inflammatory response of vascular endothelial cells. In this study, 8-bromo-cAMP, an analog of cAMP which mimicked the action of endogenous cAMP, also induced CHOP expression in HUVECs. It was reported that NF-κB pathway was important for the acidosis/GPR4-induced inflammatory gene expression in endothelial cells [21]. However, this was not the case in acidosis/GPR4-induced CHOP expression, because JSH-23, an NF-κB inhibitor, didn’t prevent HR-induced CHOP expression (Data not shown). These results indicated that acidosis/GPR4 might stimulate inflammatory response and ER stress/apoptosis via different pathways. Further studies employing endothelial-specific GPR4 knockout mice will help to clarify the role of GPR4 in vivo.

In conclusion, we demonstrate in this study that IR leads to an accumulation of CHOP protein in renal epithelial and endothelial cells, which plays an important role in ischemic renal injury. As for endothelial cells, to a large extent hypoxia-induced CHOP expression is a result of metabolic acidosis and is mediated by a pH sensor GPR4. These results highlight the importance of CHOP in apoptosis induction in the context of renal IRI, and suggest that GPR4 inhibitors or agents targeting CHOP expression may be promising in the treatment of ischemic renal injury.

## Supporting Information

Figure S1 Confirmation of bone marrow replacement by polymerase chain reaction. Bone marrow transplantation was conducted 30 days before renal IR procedures. After all the experiments were done, the mice were sacrificed and liver, kidney, spleen and bone marrow samples were subjected to a routine PCR genotyping. Primer sequences were as follows: CHOP-P1: ATGCCCTTACCTATCGTG, CHOP-P2: AACGCCAGGGTTTTCCCAGTCA, CHOP-P3: GCAGGGTCAAGAGTAGTG. These primers produced fragments of 544 bp in wild-type tissues (WT) and 320 bp in CHOP^−/−^ tissues (Δ).
(TIF)
