# Supplementary figures and images for: Ischemia/Reperfusion-Induced CHOP Expression Promotes Apoptosis and Impairs Renal Function Recovery: The Role of Acidosis and GPR4
Source: PLoS One. 2014 Oct 24;9(10):e110944. doi: 10.1371/journal.pone.0110944 (PMC4208823; doi:10.1371/journal.pone.0110944)

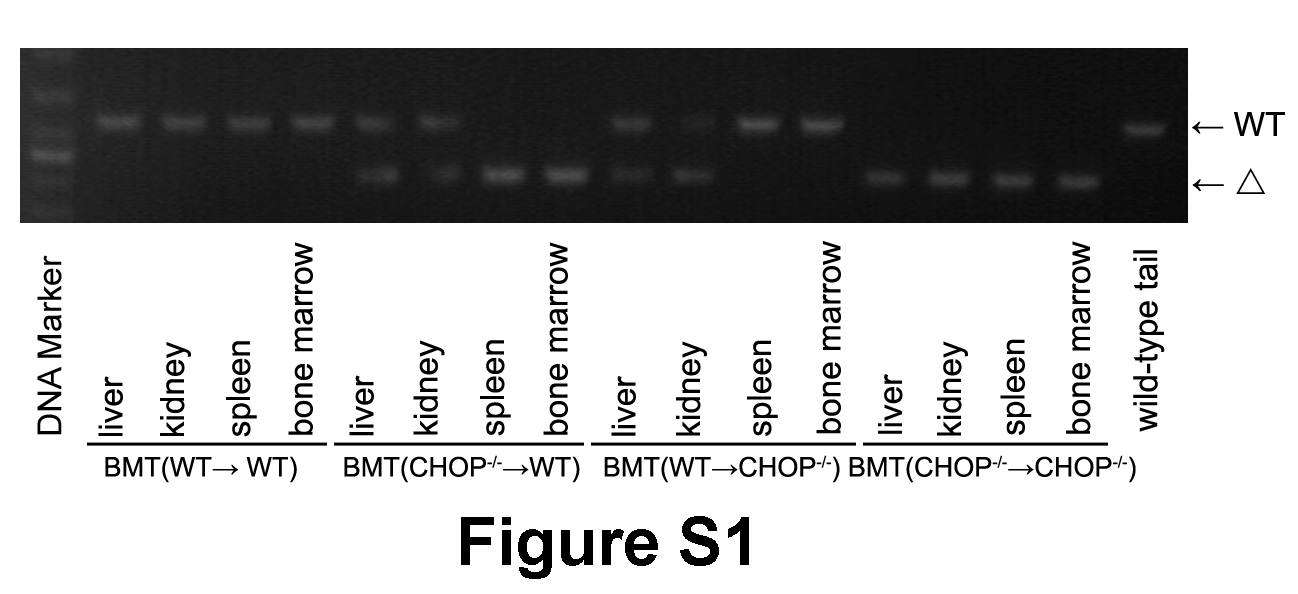

Supplement: Figure S1 — Confirmation of bone marrow replacement by polymerase chain reaction. Bone marrow transplantation was conducted 30 days before renal IR procedures. After all the experiments were done, the mice were sacrificed and liver, kidney, spleen and bone marrow samples were subjected to a routine PCR genotyping. Primer sequences were as follows: CHOP-P1: ATGCCCTTACCTATCGTG, CHOP-P2: AACGCCAGGGTTTTCCCAGTCA, CHOP-P3: GCAGGGTCAAGAGTAGTG. These primers produced fragments of 544 bp in wild-type tissues (WT) and 320 bp in CHOP−/− tissues (Δ). (TIF) [file pone.0110944.s001.tif]
